# Supplementary material for: Effect of 12-Week Daily Intake of the High-Lycopene Tomato (Solanum Lycopersicum), a Variety Named “PR-7”, on Lipid Metabolism: A Randomized, Double-Blind, Placebo-Controlled, Parallel-Group Study
Source: Nutrients. 2019 May 25;11(5):1177. doi: 10.3390/nu11051177 (PMC6566250; doi:10.3390/nu11051177)
Supplement: Supplementary file 1 [file nutrients-11-01177-s001.pdf]

Supplementary

Table S1. Nutrients from the subjects' daily diet over the past month at each visit

		**Week 0**	**ΔWeek 4**	**ΔWeek 8**	**ΔWeek 12**
Calorie (kcal)	Placebo (n = 41)	1873.1 ± 363.5	−26.1 ± 286.3	−81.7 ± 197.0	−110.7 ± 211.5
	Active (n = 33)	1726.0 ± 372.5	−51.3 ± 202.6	−36.2 ± 295.6	−43.7 ± 258.7
	p^a	0.091	0.66	0.43	0.22
Protein (g)	Placebo (n = 41)	68.3 ± 20.2	−0.7 ± 10.9	−3.5 ± 8.7	−4.0 ± 10.7
	Active (n = 33)	59.9 ± 12.5	−1.0 ± 9.7	−1.6 ± 11.2	−1.1 ± 12.3
	p^a	0.032*	0.91	0.45	0.29
Lipid (g)	Placebo (n = 41)	64.0 ± 19.0	−1.2 ± 13.3	−3.2 ± 9.8	−3.8 ± 11.1
	Active (n = 33)	60.5 ± 18.6	−4.1 ± 9.2	−2.2 ± 13.0	−2.5 ± 11.9
	p^a	0.42	0.29	0.71	0.63
Carbohydrate (g)	Placebo (n = 41)	242.1 ± 38.7	−3.3 ± 38.7	−9.8 ± 28.5	−15.1 ± 27.2
	Active (n = 33)	226.7 ± 49.8	−2.4 ± 28.8	−3.5 ± 43.0	−5.2 ± 29.2
	p^a	0.14	0.92	0.45	0.14
Dietary fiber (g)	Placebo (n = 41)	13.0 ± 3.9	−0.2 ± 2.3	−0.9 ± 2.3	−0.7 ± 2.6
	Active (n = 33)	12.0 ± 3.3	−0.4 ± 2.6	−0.5 ± 2.6	−1.0 ± 2.5
	p^a	0.24	0.79	0.55	0.57
Sodium chloride (g)	Placebo (n = 41)	9.3 ± 3.2	−1.0 ± 2.5	−1.1 ± 2.3	−1.2 ± 3.0
	Active (n = 33)	8.7 ± 2.6	−0.9 ± 1.9	−0.8 ± 2.0	−1.2 ± 1.7
	p^a	0.43	0.93	0.65	0.93

Values are shown as the mean ± standard deviation. ΔWeek 4: changes in values from baseline to week 4; ΔWeek 8: changes in values from baseline to week 8; ΔWeek 12: changes in values from baseline to week 12. Student's t-test was used for data analysis. *$p < 0.05$ vs. placebo group.

Table S2. Vital signs, body composition, complete blood count, liver function, renal function, and blood glucose profiles.

		**Week 0**	**ΔWeek 4**	**ΔWeek 8**	**ΔWeek 12**
SBP (mmHg)	Placebo (n = 49)	118.1 ± 16.1	−4.6 ± 10.4	−3.0 ± 12.0	−3.4 ± 9.4
	Active (n = 49)	119.2 ± 17.5	−3.8 ± 10.0	−1.2 ± 10.2	−2.5 ± 8.8
	p^a	0.75	0.71	0.44	0.63
DBP (mmHg)	Placebo (n = 49)	75.1 ± 10.4	−3.4 ± 6.5	−1.7 ± 7.3	−2.7 ± 6.4
	Active (n = 49)	75.8 ± 11.5	−1.8 ± 5.2	−1.1 ± 6.0	−1.0 ± 5.5
	p^a	0.78	0.20	0.64	0.17
Pulse rate (bpm)	Placebo (n = 49)	66.8 ± 10.6	2.7 ± 7.6	0.8 ± 6.3	2.0 ± 6.8
	Active (n = 49)	68.7 ± 10.3	2.5 ± 7.1	1.4 ± 8.3	0.8 ± 8.7
	p^a	0.37	0.88	0.70	0.46
BW (kg)	Placebo (n = 49)	57.2 ± 9.5	−0.2 ± 0.6	−0.2 ± 0.6	−0.3 ± 0.9
	Active (n = 49)	58.7 ± 11.8	−0.1 ± 0.7	−0.1 ± 0.8	0.1 ± 1.0
	p^a	0.49	0.55	0.37	0.095
BFR (%)	Placebo (n = 49)	26.1±6.5	−0.2 ± 0.8	−0.6 ± 1.0	−0.2 ± 1.0
	Active (n = 49)	27.3±5.9	−0.7 ± 0.8	−0.6 ± 0.9	−0.2 ± 0.9
	p^a	0.34	0.001**	0.97	0.83
BMI (kg/m^2)	Placebo (n = 49)	21.7 ± 2.6	−0.1 ± 0.2	−0.1 ± 0.2	−0.1 ± 0.4
	Active (n = 49)	22.2 ± 3.0	0.0 ± 0.3	0.0 ± 0.3	0.0 ± 0.4
	p^a	0.38	0.52	0.30	0.10
WBC (×10^3/μl)	Placebo (n = 49)	5.1 ± 1.1	0.2 ± 0.7	0.0 ± 0.7	0.2 ± 0.6
	Active (n = 49)	5.1 ± 1.3	0.1 ± 0.7	0.0 ± 0.8	0.0 ± 0.8
	p^a	0.85	0.65	0.70	0.36
RBC (×10^4/μl)	Placebo (n = 49)	466.6 ± 36.7	0.1 ± 17.1	−8.8 ± 20.3	−6.3 ± 28.7
	Active (n = 49)	468.8 ± 45.2	−3.5 ± 16.7	−9.6 ± 20.4	−7.7 ± 18.9
	p^a	0.79	0.31	0.85	0.79
Hb (g/dl)	Placebo (n = 49)	14.0 ± 1.4	0.0 ± 0.5	−0.2 ± 0.6	−0.1 ± 0.7
	Active (n = 49)	14.0 ± 1.4	−0.1 ± 0.5	−0.3 ± 0.5	−0.2 ± 0.5
	p^a	0.85	0.18	0.52	0.40

Ht (%)	Placebo (n = 49)	43.3 ± 3.7	0.0 ± 1.8	−1.2 ± 2.0	−0.6 ± 2.6
	Active (n = 49)	43.2 ± 3.7	−0.4 ± 1.6	−1.2 ± 1.9	−0.8 ± 1.6
	p^a	0.84	0.21	0.93	0.75
Plt (×10⁴/μl)	Placebo (n = 49)	24.6 ± 4.5	−0.3 ± 2.1	−0.6 ± 2.1	−0.1 ± 2.2
	Active (n = 49)	24.1 ± 4.8	0.4 ± 2.0	0.5 ± 2.2	0.6 ± 2.4
	p^a	0.63	0.095	0.018*	0.16
AST (U/l)	Placebo (n = 49)	21.1 ± 5.5	−0.3 ± 3.2	−0.6 ± 4.7	−0.9 ± 4.5
	Active (n = 49)	20.3 ± 3.6	0.9 ± 2.5	0.1 ± 2.3	−0.2 ± 2.7
	p^a	0.382	0.053	0.368	0.321
ALT (U/l)	Placebo (n = 49)	18.6 ± 7.3	−1.3 ± 4.4	−1.3 ± 6.0	−1.1 ± 6.6
	Active (n = 49)	17.7 ± 5.2	1.1 ± 4.7	0.1 ± 3.7	−0.2 ± 3.8
	p^a	0.47	0.012*	0.19	0.39
γ-GTP (U/l)	Placebo (n = 49)	27.2 ± 17.3	−2.9 ± 9.3	−2.7 ± 8.8	−0.3 ± 10.7
	Active (n = 49)	23.6 ± 13.0	0.1 ± 7.1	0.1 ± 5.7	1.6 ± 8.7
	p^a	0.25	0.082	0.075	0.35
ALP (U/l)	Placebo (n = 49)	214.0 ± 57.7	−1.1 ± 19.6	−6.7 ± 21.6	−4.2 ± 21.0
	Active (n = 49)	189.3 ± 45.9	−1.2 ± 15.5	−2.6 ± 16.3	−2.3 ± 18.7
	p^a	0.021*	0.99	0.32	0.65
LDH (U/l)	Placebo (n = 49)	190.4 ± 31.1	−8.3 ± 21.4	− 11.9 ± 19.5	−16.6 ± 19.0
	Active (n = 49)	192.4 ± 30.8	−2.8 ± 20.1	−10.6 ± 21.5	−12.9 ± 21.8
	p^a	0.75	0.20	0.76	0.38
BUN (mg/dL)	Placebo (n = 49)	13.2 ± 3.3	0.9 ± 2.5	0.7 ± 2.8	1.1 ± 2.1
	Active (n = 49)	14.0 ± 2.7	0.3 ± 2.5	0.1 ± 2.5	0.6 ± 2.4
	p^a	0.21	0.26	0.32	0.27
CRE (mg/dL)	Placebo (n = 49)	0.8 ± 0.1	0.0 ± 0.0	0.0 ± 0.1	0.0 ± 0.1
	Active (n = 49)	0.8 ± 0.1	0.0 ± 0.1	0.0 ± 0.1	0.0 ± 0.1
	p^a	0.36	0.62	0.63	0.35
UA (mg/dL)	Placebo (n = 49)	5.1 ± 1.1	0.1 ± 0.6	0.2 ± 0.6	0.3±0.7
	Active (n = 49)	5.5 ± 1.2	0.2 ± 0.5	0.1 ± 0.5	0.2±0.6
	p^a	0.13	0.89	0.50	0.56
FPG (mg/dL)	Placebo (n = 49)	88.0 ± 7.0	1.7 ± 4.3	1.4 ± 3.8	0.4 ± 4.5
	Active (n = 49)	87.8 ± 8.7	2.1 ± 5.3	0.6 ± 3.2	−0.7 ± 4.3
	p^a	0.91	0.71	0.25	0.24
HbA1c (%)	Placebo (n = 49)	5.5 ± 0.3	−0.1 ± 0.3	0.0 ± 0.2	−0.2 ± 0.2
	Active (n = 49)	5.6 ± 0.4	−0.1 ± 0.2	0.0 ± 0.1	−0.2 ± 0.2
	p^a	0.15	0.86	0.89	0.93
HOMA-IR	Placebo (n = 49)	0.8 ± 0.5	0.1 ± 0.3	0.1 ± 0.4	0.1 ± 0.3
	Active (n = 49)	0.8 ± 0.4	0.1 ± 0.4	0.1 ± 0.4	0.1 ± 0.3
	p^a	0.86	0.50	0.73	0.73

Values are shown as the mean ± standard deviation. Student's t-test was performed. * $p^a < 0.05$ vs. placebo group. ΔWeek: changes in values from baseline to week 4; ΔWeek 8: changes in values from baseline to week 8; ΔWeek 12: changes in values from baseline to week 12; SBP: systolic blood pressure; DBP: diastolic blood pressure; bpm: beats per minute; BW: body weight; BFR: body fat rate; BMI: body mass index; WBC: white blood cell; RBC: red blood cell; Hb: hemoglobin; Ht: hematocrit; Plt: platelet; AST: aspartate aminotransferase; ALT: alanine aminotransferase; γ-GTP: gamma-glutamyl transpeptidase; ALP: alkaline phosphatase; LDH: lactate dehydrogenase; BUN: blood urea nitrogen; CRE: creatinine; UA: uric acid; FPG; fasting plasma glucose; HbA1c: hemoglobin A1c; HOMA-IR: homeostasis model assessment of insulin resistance.
